# Gaming behaviour with Pokémon GO and physical activity: A preliminary study with medical students in Thailand

**DOI:** 10.1371/journal.pone.0199813

**Published:** 2018-06-29

**Authors:** Apichai Wattanapisit, Udomsak Saengow, Chirk Jenn Ng, Sanhapan Thanamee, Nonthakorn Kaewruang

**Affiliations:** 1 School of Medicine, Walailak University, Thasala, Nakhon Si Thammarat, Thailand; 2 Center of Excellence in Health System and Medical Research, Walailak University, Thasala, Nakhon Si Thammarat, Thailand; 3 Department of Primary Care Medicine, Faculty of Medicine, University of Malaya, Kuala Lumpur, Malaysia; 4 Thasala Hospital, Thasala, Nakhon Si Thammarat, Thailand; CHEO Research Institute, CANADA

## Abstract

Pokémon GO becomes the most rapidly downloaded mobile application in history. This study aimed to determine the physical activity of medical students, who played Pokémon GO, and the change in their use of Pokémon GO and physical activity over time. An observational study was conducted. Physical activity was measured by using self-administered questionnaires at baseline (phase 0), 1 month (phase 1) and 3 months (phase 2) post-Pokémon GO download. The changes in physical activity (phase 0 to 1 and phase 1 to 2) were analysed using Wilcoxon Signed Ranked test. The trend (3-point analysis) of physical activity from phase 0, 1 to 2 were analysed using Friedman’s test. The relationship between physical activity and time spent gaming was analysed by using Spearman’s rank correlation. Twenty-six participants (mean age 22.04±1.70 years) participated in the study. There was no statistically significant change in physical activity during the three-month period (p = 0.45). Only 11 participants (42.3%) were still playing Pokémon GO 3 months after download. The key reasons for playing game were ‘have fun’ and ‘pass time/boredom’. The most common commuting mode to play the game was walking; some drove a car or motorcycle while playing the game. There was no correlation between physical activity and time spent gaming. This study highlights how the lack of sustainability of the game and the motivation behind using Pokémon GO as a game rather than a physical activity app may have undermined the potential of using the game to improve physical activity. Further studies need to explore the reasons for the lack of sustainability and how to combine fun with behavioural change.

## Introduction

Physical inactivity is an important health risk that contributes to 3.2 million deaths and 69.3 million disability-adjusted life year (DALYs) globally each year.[1] Moreover, it is a risk factor for major non-communicable diseases (NCDs), including coronary heart disease, type 2 diabetes, breast cancer, and colon cancer.[2]

To effectively increase physical activity (PA) at a population level requires multi-sectoral, multi-disciplinary multi-strategy-based approaches.[3–5] The expansion of mobile devices and computational intelligence has led to the development of many approaches of motivating people to participate in and monitoring their PA.[6] Therefore, the use of digital technologies has been increasingly recognised as a potentially effective intervention, to increase PA, particularly among the younger people who are leading a sedentary lifestyle.[7–9] For example, playing active video games has been found to increase PA levels in both children and adults [10, 11] and PA mobile phone games may be an important element effecting behavioural change in PA interventions.[12]

Pokémon GO is an augmented reality game which involves virtual elements that build upon the existing environment.[13] The game requires players to physically move to collect Pokémon (game characters) and walk in the real world for 2, 5 or 10 km to hatch a Pokémon egg.[14, 15] Pokémon GO was first released in July 2016 and launched in Asia in August 2016.[16, 17] It becomes the most rapidly downloaded mobile application in history.[18, 19] In view of its PA-related game patterns and its popularity, we hypothesised that playing Pokémon GO could increase and potentially maintain PA of game players. One study has already shown that Pokémon GO significantly increased PA of game players in the first 30 days of playing the game.[20]

In Thailand, physical inactivity is on the rise, particularly among young adults.[21] This correlates with the rising incidence of obesity and diabetes in the younger population.[22–25] A recent study of a group of Thai medical students found that more than half (50.5%) of them with a mean age of 20.93±1.82 were physically inactive.[26] However, motivating young people to engage in regular PA remains challenging.[27, 28] This study aimed to investigate the gaming behaviour of a group of medical students in Southern Thailand who were actively playing Pokémon GO, and the change in their PA. Moreover, the present study focused on the sustainability of the game over time.

## Materials and methods

### Study design

We conducted an observational study. The baseline data (phase 0) was obtained from the PA survey conducted in 2015 with medical students of Walailak University.[26] Phase 1 data was collected in September 2016, 1 month after downloading and playing Pokémon GO. The data included: demographic data, patterns of and reasons for playing Pokémon GO, game-related injuries, PA and sedentary time. Phase 2 data was collected three months after downloading the game and they were: patterns of and reasons for playing Pokémon GO, game-related injuries, PA and sedentary time. The online questionnaires were used to collect all the data in phase 1 and phase 2.

### Setting and participants

The study was conducted in the three campuses of School of Medicine, Walailak University, in Southern Thailand (Nakhon Si Thammarat, Trang and Phuket). Preclinical medical students (years 1–3) studied at Nakhon Si Thammarat campus while the clinical students (years 4–6) did their clinical rotations at Trang or Phuket campus. All medical students who participated in the previous PA survey were eligible to participate in this study, except for the year 1 and 4 students.[26] The year 1 students were excluded because we did not have their baseline data at Phase 0 while the year 4 students were excluded because there was a transition from preclinical to clinical year, which was a potential confounding factor.[26] A total of 189 medical students (years 2, 3, 5 and 6) met these inclusion criteria. We asked the medical students who had played Pokémon GO at least 1 month to participate in the study. Students with disabilities or medical conditions which limited their ability of PA, including movement disorder; muscular weakness; heart disease; uncontrolled pulmonary disease; and uncontrolled medical conditions, were excluded. All the participants from phase 1 were asked to complete the questionnaire in phase 2. We reminded them through Facebook or telephone.

### Research instruments

The phase 1 questionnaire consisted of three parts: participant profiles (level of education, sex, birth date, body weight and height, student’s allowance, place of living and underlying illnesses); patterns of playing Pokémon GO (time spent gaming, mode of commuting while playing game, reasons for playing Pokémon GO and game-related injuries) and information on PA and sedentary time using the Global Physical Activity Questionnaire (GPAQ) version 2 (Thai version). The phase 2 questionnaire excluded the participant profiles.

We developed the questionnaires in Thai language. All the items were initially drafted in Thai except the reasons for playing Pokémon GO, which were adapted from an existing questionnaire [29]. The items were translated and back-translated by two researchers (AW and ST). The questionnaires were assessed by two external PA experts for content validation. The finalised version of the questionnaires was created online via Google Forms. A pilot study was conducted with four students to determine the face validity of the questionnaires as well as to test the study flow and recruitment strategy.

### Data analysis

Participants’ demographic characteristics, reasons for and patterns of playing games, and injury pattern were analysed using percentages for categorical variables, and mean with standard deviation (SD) for continuous variables. PA participation and sedentary time were measured at baseline (phase 0, non-Pokémon GO players), 1 month (phase 1, Pokémon GO players) and 3 months (phase 2, both recent and former players) post-Pokémon GO download. The PA participation, including moderate- to vigorous-intensity PA, was presented as energy expenditure using the following formula: energy expenditure = [4 MET X time of moderate PA (minutes/week)] + [8 MET X time of vigorous PA (minutes/week)].[30] The sedentary time was adjusted and presented as minutes/day.

The changes in PA and sedentary time from phase 0 to phase 1 as well as from phase 1 to phase 2 were analysed using Wilcoxon Signed Ranked test. The trend (3-point analysis) of PA and sedentary time from phase 0, 1 to 2 were analysed using Friedman’s test. The relationships between PA, sedentary time and time spent on gaming were analysed using Spearman’s rank correlation. The relationship was considered statistically significant when p<0.05.

### Ethics approval

Ethical approval was obtained from the Human Research Ethics Committee Walailak University (protocol number 061/2016). All medical students were above 18 years old (legal age for consent in Thailand) and their participation was entirely voluntary. Information about the study was provided on the first screen of the online questionnaire and the informed consent was taken by asking the participants to indicate their agreement to participate by ticking a checkbox.

## Results

### Demographic characteristics of Pokémon GO players

Twenty-six medical students, who played Pokémon GO, participated in the study; 65.4% (n = 17) were clinical students; and 73.1% (n = 19) were men. The mean age was 22.04±1.70 years. About half (53.8%, n = 14) were of a healthy weight.[31] Most participants (92.3%, n = 24) had allowance between 5000 and 14999 Baht/month (USD142.69 and 428.05/month). The majority of the participants (96.2%, n = 25) lived on campus (Table 1).

**Table 1:** Demographic characteristics of Pokémon GO players.

Demographic characteristics	Phase 1 (n = 26) n (%)	Phase 2 (n = 11) n (%)
Age (year) mean (SD)	22.04 (1.70)	22.20 (1.55)
Sex		
• Male	19 (73.1)	8 (72.7)
• Female	7 (26.9)	3 (27.3)
Weight status		
• Underweight	5 (19.2)	2 (18.2)
• Healthy weight	14 (53.8)	5 (45.4)
• Overweight	5 (19.2)	2 (18.2)
• Obese	2 (7.7)	2 (18.2)
Allowance (Baht/month)^a^		
• <5000	1 (3.8)	(9.1)
• 5000–14999	24 (92.3)	10 (90.9)
• ≥15000	1 (3.8)	0
Place of living		
• On campus	25 (96.2)	10 (90.9)
Underlying illness		
• Yes^b^	1 (3.8)	0

Data presented as mean (SD) or n (%).

^a^ 35.04 Baht = US$1.

^b^ Bone cyst

### Physical activity participation and sedentary time

**Fig 1:**
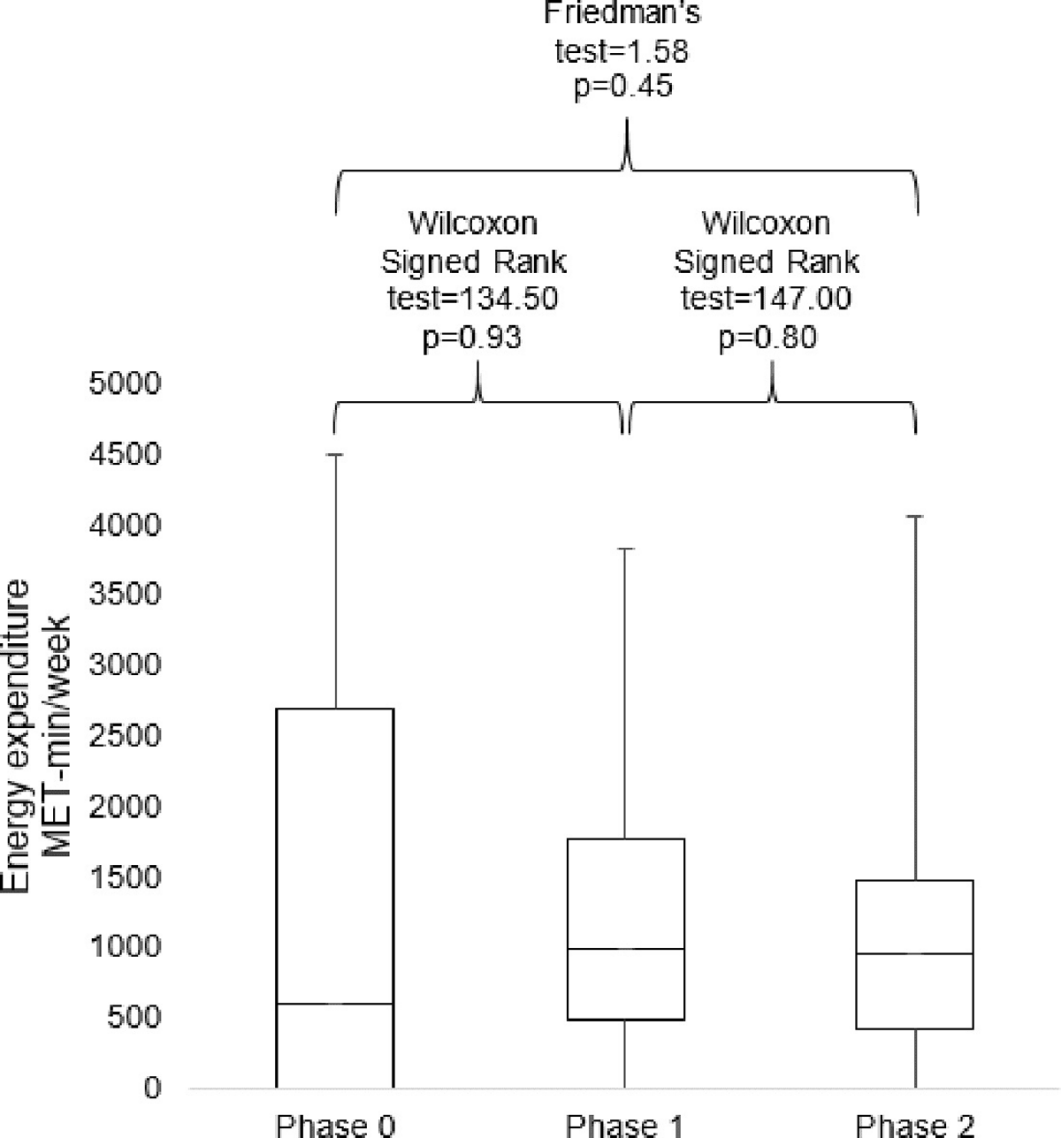
Comparisons of energy expenditure among participants in phase 0, 1 and 2.

In phase 0, the median energy expenditure from moderate- to vigorous-intensity PA was 600 MET-min/week (IQR 0–2700). In phase 1 and phase 2, the median energy expenditure were 996 MET-min/week (IQR 490–1770) and 960 MET-min/week (IQR 420–1470), respectively. There was no statistically significant difference in energy expenditure between phase 0 and phase 1 (p = 0.93), as well as phase 1 and phase 2 (p = 0.80). The 3-point analysis of PA from phase 0, 1 to 2 did not show any significant change (p = 0.45) (Fig 1).

**Fig 2:**
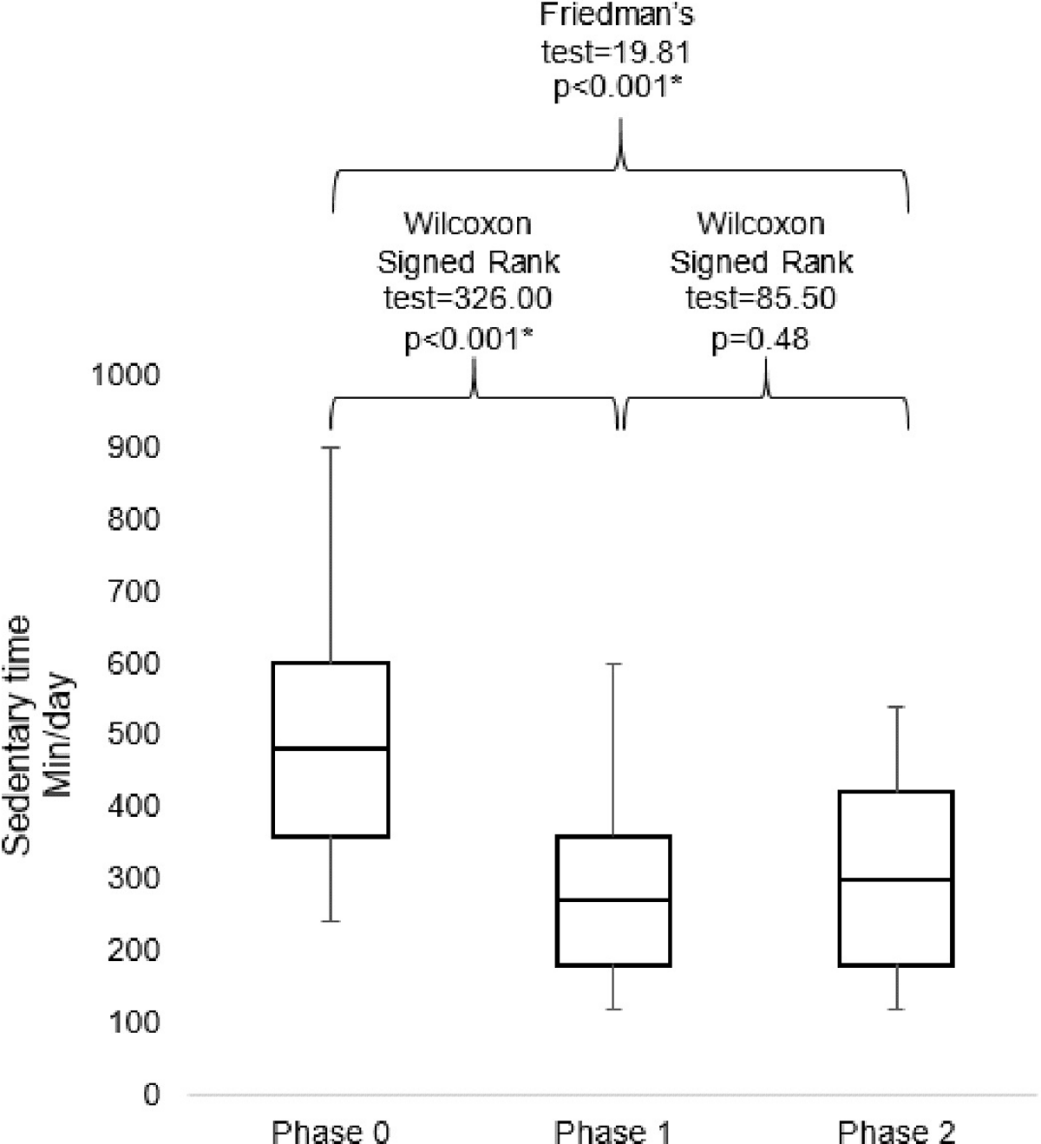
Comparisons of sedentary time among participants in phase 0, 1 and 2.

The median sedentary time in phase 0 was 480 min/week (IQR 360–600), and it decreased significantly to 270 min/week (IQR 180–360) (p<0.001) in phase 1. The median sedentary time in phase 2 was 300 min/week (IQR 180–420). The change in sedentary time from phase 1 to 2 was not significant (p = 0.48). Sedentary time was changed significantly through the 3-point analysis (p<0.001) (Fig 2).

### Patterns of and reasons for playing Pokémon GO and game-related injuries

In phase 1, a total of 26 medical students played Pokémon GO, of which majority (92.3%, n = 24) played the game at least 3–5 days/week. Overall, the participants spent 360 min/week (IQR 120–630) playing the game. The most frequently reported reasons for playing game were: ‘have fun’ (84.6%, n = 22), ‘pass time/boredom’ (80.9%, n = 21), and ‘relax/de-stress’ (76.9%, n = 20) (Table 2). Most participants responded to the game conditions by walking (88.5%, n = 23), followed by riding a car (61.5%, n = 18), riding a motorcycle (50%, n = 13), running (23.1%, n = 6) and cycling (11.5%, n = 3). There were no game-related injuries reported in phase 1.

**Table 2:** Patterns of and reasons for playing Pokémon GO.

Playing Pokémon GO	Phase 1 (n = 26)	Phase 2 (n = 11)
Frequency, n (%)		
• 1–2 days/week	2 (7.7)	7 (63.6)
• 3–5 days/week	13 (50.0)	4 (36.4)
• 6–7 days/week	11 (42.3)	0
Time spent gaming (minute/week), median (IQR)	360 (120–630)	60 (60–80)
Reason, n (%)		
• Have fun	22 (84.6)	6 (54.5)
• Pass time/boredom	21 (80.8)	9 (81.8)
• Relax/de-stress	20 (76.9)	9 (81.8)
• Social interaction	12 (46.2)	1 (9.1)
• Be challenged	10 (38.5)	3 (27.3)
• Feel excitement	8 (30.8)	1 (9.1)
• Exercise	7 (26.9)	2 (18.2)
• Do the impossible	2 (7.7)	0
• Learn	2 (7.7)	0
Commuting mode, n (%)		
• Walking	23 (88.5)	8 (100)
• Riding a car	18 (61.5)	5 (45.4)
• Riding a motorcycle	13 (50.0)	5 (45.4)
• Running	6 (23.1)	1 (18.2)
• Cycling	3 (11.5)	1 (9.1)

IQR, interquartile range

In phase 2, out of the 26 participants, only 11 medical students were still playing Pokémon GO (Table 1 shows the demographic characteristics) and most were playing the game 1–2 days/week (63.6%, n = 7), and 36.4% (n = 4) spent 3–5 days/week. The median time spent gaming was 60 min/week (IQR 60–80). The most common reasons for playing game were ‘pass time/boredom’ (81.8%, n = 9) and ‘relax/de-stress’ (81.8%, n = 9) (Table 2). All players walked; 45.4% (n = 5) rode a car; 45.4% (n = 5) rode a motorcycle; 18.2% (n = 2) ran and 9.1% (n = 1) cycled to respond to the game conditions. There was no game-related injuries among the game players.

### Relationships between energy expenditure, sedentary time and time spent gaming

**Fig 3:**
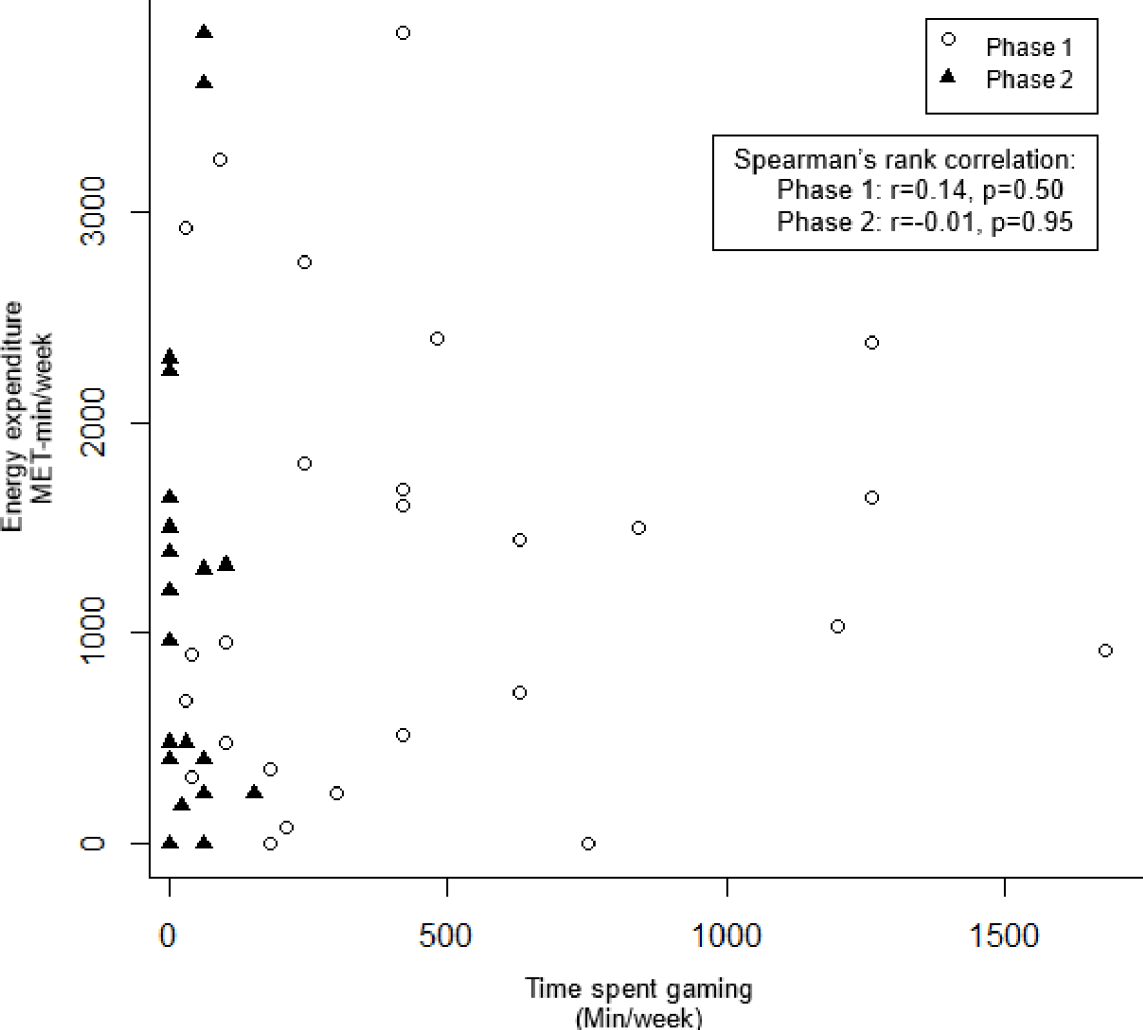
Relationships between energy expenditure and time spent gaming.

**Fig 4:**
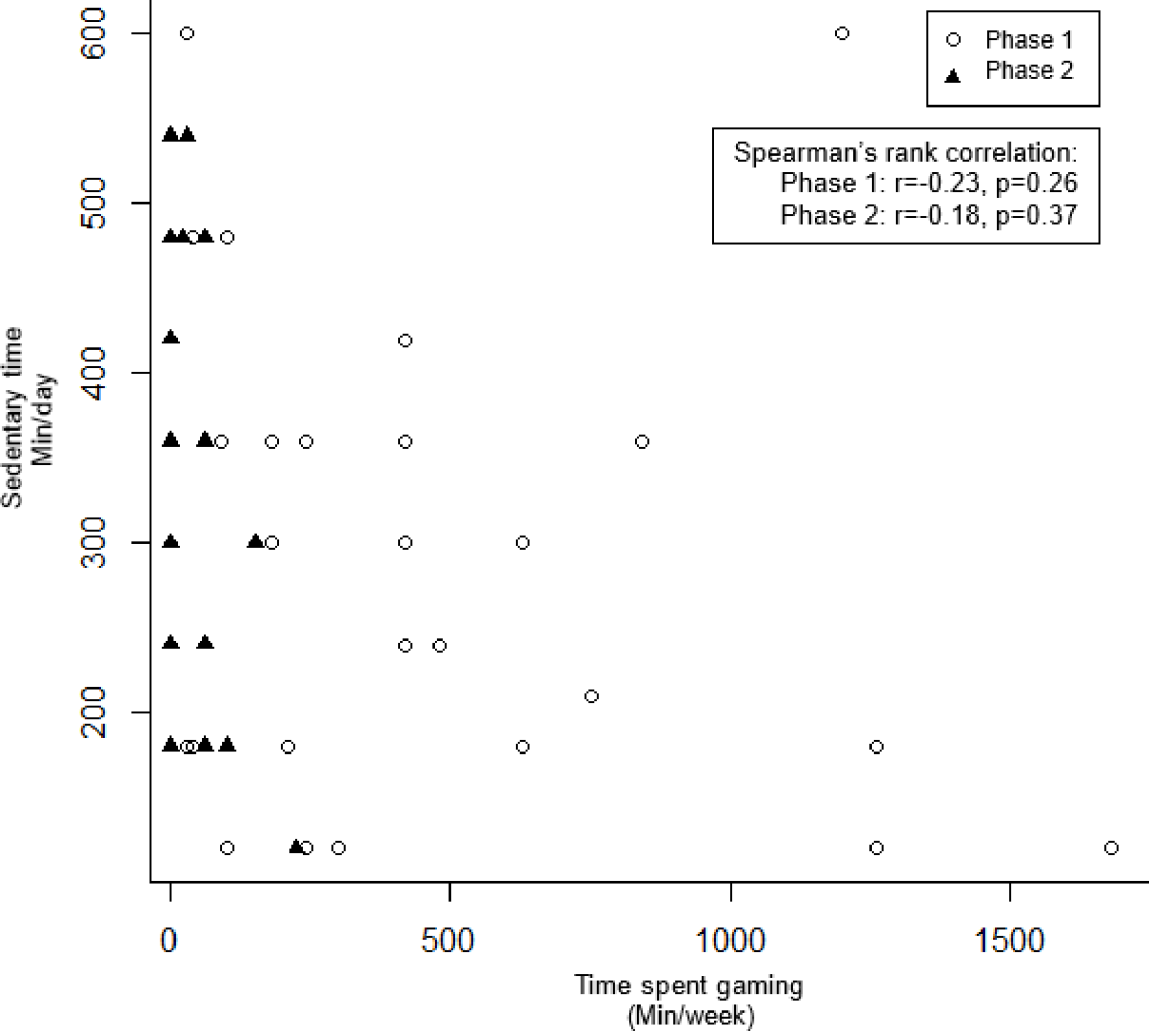
Relationships between sedentary time and time spent gaming.

Fig 3 shows that there were no significant relationships between energy expenditure and time spent gaming among game players in both phase 1 (p = 0.50) and phase 2 (p = 0.95). Similarly, there was no correlation between sedentary time and time spent gaming in both phase 1 (p = 0.26) and phase 2 (p = 0.37) (Fig 4).

## Discussion

The findings from this study contradict those found in some Pokémon GO studies. The number of students who played Pokémon GO dropped significantly over three months and the game did not seem to change the participants’ level and duration of physical activities. In addition, there were no correlations between time spent gaming and energy expenditure. This challenged the hypothesis that Pokémon GO was effective in improving PA among those playing the game. However, there was a significant decrease in sedentary time over three months, although it was not correlated to gaming time.

A USA study investigated the effect of Pokémon GO on PA via wearable PA trackers among 792 Pokémon GO players. The study found that Pokémon GO led to significantly increased walking by 1473 steps a day on average, over a period of 30 days. There was also a dose-response relationship between Pokémon GO and PA.[20] The same trend was found in the study by Howe et al. where Pokémon GO users recorded 955 additional steps per day during the first week of installation of the game. However, the number of daily steps dropped significantly by the sixth week after installation.[32] Studies among college students also found Pokémon GO might be an effective tool to increase PA.[33–35] In contrast, a cross-sectional study conducted among university students in Hong Kong did not find any significant difference in PA levels between Pokémon GO players and non-players at 4 weeks after the releasing of the game.[36]

Most previous studies focused on the influence of Pokémon GO on PA over the first 4–6 weeks of playing the game.[20, 32, 36] While some studies found positive effects on PA within the first few weeks after playing the game,[20, 32] others found the game to be ineffective in young and older adults.[36, 37] Our study extended the follow up period to three months to look at the sustainability of the game as well as its effect on PA. The findings found that the game might not change the participants’ PA levels among a group of university students, and the number of game players decreased significantly. This finding is important because it provides preliminary evidence on the limitation of using a game app to improve PA and that more studies need to look into the factors that influence the sustainable use of the game and how the mechanisms of behavioural change are effected via games. This observation is supported by studies that propose gaming alone might not effective; combining gaming with motivation and social support might work better.[20, 36]

Among the participants in this study, the main reason for playing Pokémon GO was not to exercise; only a quarter of the participants played the game for exercise which dropped even lower at the end of phase 2. At 3 months after downloading the game, some participants became the former players, meaning that they were not interested in playing Pokémon GO. Among the active players, all of them still walked to catch the Pokémon, however, they spent less time and frequency to play the game. Therefore, the increases in PA from the previous studies [20, 32] might be a side effect of the game.[38] Although the players were required to travel to public places to catch Pokémon and incubate Pokémon eggs,[14, 15] some players in our study travelled by taking slow-moving vehicles instead of walking. This could explain the lack of correlation between PA and time spent playing the game. In addition, although we found a significant decrease in sedentary time among the Pokémon GO players, it was not directly correlated to time spent gaming. It might be an indirect effect of the game that causing the players to stay outdoor.[36]

The strengths of this study were 1) the study provided longer term evaluations of the game usage pattern and its effect on PA from baseline to three months after downloading and playing the game, and 2) it presented patterns of and reasons for playing Pokémon GO over time, which could explain the sustainability of using the game to seek PA.

There were some limitations of the present study. First, a key limitation of the study was a small sample size. We needed 130 medical students to achieve the sample size, but we had only managed to recruit 13.8% (n = 26) of the target population. At 3 months after downloading the game, less than half of the participants were still playing the game. This limits the power of our study to detect statistical significances and associations between variables. For example, there was a big change in the PA participation between phase 0 and phase 1 (from 600 MET-min/week to 996 MET-min/week) despite not being statistically significant. While the change in sedentary time between phase 0 and phase 1 was significant. This interpretation should be aware. Reasons that can explain the cause of the small sample size and high dropout rate probably because of 1) the decreased in popularity of the Pokémon GO game during the study period, 2) the recruitment of participants which included only Pokémon GO players who played the game more than a month, and 3) the lack of Pokéstops (where players could get essential items to play the game) in rural areas, where this study was conducted. This might limit the interest and dissemination of the game.

Second, using GPAQ, a self-reporting questionnaire, to collect the PA participation may lead to the biased data collection due to the small change in PA and potential recall bias of the participants.[39, 40] More precise measurements of PA using accelerometers or performing shorter reporting spans (ecological momentary assessment) may be better suited for measuring PA in such study. Third, the lack generalisability because the study was conducted among medical students who might have a different PA patterns compared to young people in the community. Nevertheless, the findings from this study shed light on the PA and gaming behaviour of a group of Thai university students. Fourth, only Pokémon GO players were invited to participate in this study. Therefore, the difference between PA patterns of Pokémon GO players and non-Pokémon GO players could not be identified.

Safety issue has been a major concern of Pokémon GO and future augmented reality games. This is due to the risk of games-related injuries such as repetitive strain injuries [36, 41] as well as serious events due to distractions from the real world and criminal incidences.[42–44] However, our study did not report any injuries or adverse events from using Pokémon GO.

## Conclusions

Despite its potential to change PA behaviour, this study found that Pokémon GO may lack sustainability, which in turns might have affected its usefulness as a tool to improve PA. The motivation behind using Pokémon GO as a game rather than a PA app might have undermined the effectiveness of the game to improve PA. Further studies with a bigger sample size need to be conducted to explore how to combine fun with behavioural change and make it sustainable.

## Supporting information

S1 File Survey questions. (DOCX)

## References

[1] World Health Organization. Global status report on communicale diseases 2014. Geneva, Switzerland: World Health Organization; 2014.

[2] Lee IM, Shiroma EJ, Lobelo F, Puska P, Blair SN, Katzmarzyk PT. Effect of physical inactivity on major non-communicable diseases worldwide: an analysis of burden of disease and life expectancy. Lancet. 2012;380(9838):219–29. Epub 2012/07/24. doi: 10.1016/S0140-6736(12)61031-9 22818936 10.1016/S0140-6736(12)61031-9 PMC3645500

[3] Conn VS, Hafdahl AR, Mehr DR. Interventions to increase physical activity among healthy adults: meta-analysis of outcomes. Am J Public Health. 2011;101(4):751–8. doi: 10.2105/AJPH.2010.194381 21330590 10.2105/AJPH.2010.194381 PMC3052337

[4] Kahn EB, Ramsey LT, Brownson RC, Heath GW, Howze EH, Powell KE, et al The effectiveness of interventions to increase physical activity. A systematic review. Am J Prev Med. 2002;22(4 Suppl):73–107. 11985936 10.1016/s0749-3797(02)00434-8

[5] Reis RS, Salvo D, Ogilvie D, Lambert EV, Goenka S, Brownson RC. Scaling up physical activity interventions worldwide: stepping up to larger and smarter approaches to get people moving. Lancet. 2016;388(10051):1337–48. doi: 10.1016/S0140-6736(16)30728-0 27475273 10.1016/S0140-6736(16)30728-0 PMC5193005

[6] Fister I Jr, Ljubič K, Suganthan PN, Perc M, Fister I. Computational intelligence in sports: Challenges and opportunities within a new research domain. Appl Math Comput. 2015;262:178–86. https://doi.org/10.1016/j.amc.2015.04.004

[7] Dallery J, Kurti A, Erb P. A new frontier: integrating behavioral and digital technology to promote health behavior. Behav Anal. 2015;38(1):19–49. Epub 2016/06/28. doi: 10.1007/s40614-014-0017-y 27347477 10.1007/s40614-014-0017-y PMC4883489

[8] Kurti AN, Dallery J. Integrating technological advancements in behavioral interventions to promote health: unprecedented oportunities for behavior analysts. Rev Mex Anal Conducta. 2014;40(2):106–26. 25774070 PMC4358800

[9] Tate DF, Lyons EJ, Valle CG. High-tech tools for exercise motivation: use and role of technologies such as the internet, mobile applications, social media, and video games. diabetes spectrum. Diabetes Care. 2015;28(1):45–54. doi: 10.2337/diaspect.28.1.45 25717278 10.2337/diaspect.28.1.45 PMC4334081

[10] Sween J, Wallington SF, Sheppard V, Taylor T, Llanos AA, Adams-Campbell LL. The role of exergaming in improving physical activity: a review. J Phys Act Health. 2014;11(4):864–70. doi: 10.1123/jpah.2011-0425 25078529 10.1123/jpah.2011-0425 PMC4180490

[11] Foley L, Maddison R. Use of active video games to increase physical activity in children: a (virtual) reality? Pediatr Exerc Sci. 2010;22(1):7–20. 20332536 10.1123/pes.22.1.7

[12] Payne HE, Moxley VBA, MacDonald E. Health behavior theory in physical activity game apps: a content analysis. JMIR Serious Games. 2015;3(2). doi: 10.2196/games.4187 26168926 10.2196/games.4187 PMC4526993

[13] Baus O, Bouchard S. Moving from virtual reality exposure-based therapy to augmented reality exposure-based therapy: a review. Front Hum Neurosci. 2014;8 doi: 10.3389/fnhum.2014.00008 24624073 10.3389/fnhum.2014.00112 PMC3941080

[14] Anderson N, Steele J, O'Neill LA, Harden LA. Pokemon Go: mobile app user guides. Br J Sports Med. 2016 doi: 10.1136/bjsports-2016-096762

[15] Ayers JW, Leas EC, Dredze M, Allem JP, Grabowski JG, Hill L. Pokemon GO-a new distraction for drivers and pedestrians. JAMA internal medicine. 2016 doi: 10.1001/jamainternmed.2016.6274 27635638 10.1001/jamainternmed.2016.6274

[16] Rujivanarom P. Psychiatrist warns players of Pokemon Go addiction as game finally launches in Thailand. 2016 Aug 7. [cited 12 August 2016]. In: The Nation [Internet]. Available from: http://www.nationmultimedia.com/news/national/30292307

[17] Serino M, Cordrey K, McLaughlin L, Milanaik RL. Pokemon Go and augmented virtual reality games: a cautionary commentary for parents and pediatricians. Curr Opin Pediatr Curr. 2016;28(5):673–7. doi: 10.1097/mop.0000000000000409 27479151 10.1097/MOP.0000000000000409

[18] Crider M. Pokémon GO passes 100 million Play Store downloads in just a month. 2016 Aug 8. [cited 12 August 2016]. In: Android Police [Internet]. Available from: http://www.androidpolice.com/2016/08/08/pokmon-go-passes-100-million-play-store-downloads-just-month/

[19] Dillet R. Apple says Pokémon Go is the most downloaded app in a first week ever. 2016 Jul 22. [cited 2 November 2016 ]. In: Techcrunch [Internet]. Available from: https://techcrunch.com/2016/07/22/apple-says-pokemon-go-is-the-most-downloaded-app-in-its-first-week-ever/

[20] Althoff T, White RW, Horvitz E. Influence of Pokemon Go on physical activity: study and implications. J Med Internet Res. 2016;18(12):e315 doi: 10.2196/jmir.6759 27923778 10.2196/jmir.6759 PMC5174727

[21] Topothai T, Chandrasiri O, Liangruenrom N, Tangcharoensathien V. Renewing commitments to physical activity targets in Thailand. Lancet. 2016;388(10051):1258–60. doi: 10.1016/S0140-6736(16)30929-1 27475272 10.1016/S0140-6736(16)30929-1

[22] Banks E, Lim L, Seubsman SA, Bain C, Sleigh A. Relationship of obesity to physical activity, domestic activities, and sedentary behaviours: cross-sectional findings from a national cohort of over 70,000 Thai adults. BMC Public Health. 2011;11:762 doi: 10.1186/1471-2458-11-762 21970620 10.1186/1471-2458-11-762 PMC3204261

[23] Gill T. Young people with diabetes and obesity in Asia: a growing epidemic. Diabetes Voice. 2007;52:20–2.

[24] Ramachandran A, Snehalatha C. Rising burden of obesity in Asia. J Obes. 2010;2010 doi: 10.1155/2010/868573 20871654 10.1155/2010/868573 PMC2939400

[25] Ramachandran A, Snehalatha C, Shetty AS, Nanditha A. Trends in prevalence of diabetes in Asian countries. World J Diabetes. 2012;3(6):110–7. doi: 10.4239/wjd.v3.i6.110 22737281 10.4239/wjd.v3.i6.110 PMC3382707

[26] Wattanapisit A, Fungthongcharoen K, Saengow U, Vijitpongjinda S. Physical activity among medical students in Southern Thailand: a mixed methods study. BMJ open. 2016;6(9):e013479 doi: 10.1136/bmjopen-2016-013479 27678548 10.1136/bmjopen-2016-013479 PMC5051498

[27] Molanorouzi K, Khoo S, Morris T. Motives for adult participation in physical activity: type of activity, age, and gender. BMC Public Health. 2015;15(1):66 doi: 10.1186/s12889-015-1429-7 25637384 10.1186/s12889-015-1429-7 PMC4314738

[28] Poobalan AS, Aucott LS, Clarke A, Smith WCS. Physical activity attitudes, intentions and behaviour among 18–25 year olds: A mixed method study. BMC Public Health. 2012;12(1):640 doi: 10.1186/1471-2458-12-640 22892291 10.1186/1471-2458-12-640 PMC3490897

[29] Brand JE, Todhunter S. Digital Australia Report 2016. Eveleigh, NSW: IGEA; 2015.

[30] Word Health Organization. Global Physical Activity Questionnaire (GPAQ) analysis guide Word Health Organization. [cited 13 August 2016]. In: Word Health Organization [Internet]. Available from: http://www.who.int/chp/steps/resources/GPAQ_Analysis_Guide.pdf

[31] Kanazawa M, Yoshiike N, Osaka T, Numba Y, Zimmet P, Inoue S. Criteria and classification of obesity in Japan and Asia-Oceania. World Rev Nutr Diet. 2005;94:1–12. doi: 10.1159/000088200 16145245 10.1159/000088200

[32] Howe KB, Suharlim C, Ueda P, Howe D, Kawachi I, Rimm EB. Gotta catch'em all! Pokemon GO and physical activity among young adults: difference in differences study. BMJ. 2016;355:i6270 doi: 10.1136/bmj.i6270 27965211 10.1136/bmj.i6270 PMC5154977

[33] Barkley JE, Lepp A, Glickman EL. "Pokemon Go!" May Promote Walking, Discourage Sedentary Behavior in College Students. Games Health J. 2017;6(3):165–70. doi: 10.1089/g4h.2017.0009 28628384 10.1089/g4h.2017.0009

[34] Marquet O, Alberico C, Adlakha D, Hipp JA. Examining Motivations to Play Pokemon GO and Their Influence on Perceived Outcomes and Physical Activity. JMIR Serious Games. 2017;5(4):e21 doi: 10.2196/games.8048 29066423 10.2196/games.8048 PMC5676026

[35] Marquet O, Alberico C, Hipp AJ. Pokémon GO and physical activity among college students. A study using Ecological Momentary Assessment. Comput Human Behav. 2018;81:215–22.

[36] Wong FY. Influence of Pokemon Go on physical activity levels of university players: a cross-sectional study. Int J Health Geogr. 2017;16(1):8 doi: 10.1186/s12942-017-0080-1 28228102 10.1186/s12942-017-0080-1 PMC5322678

[37] Rasche P, Schlomann A, Mertens A. Who Is Still Playing Pokemon Go? A Web-Based Survey. JMIR Serious Games. 2017;5(2):e7 doi: 10.2196/games.7197 28381393 10.2196/games.7197 PMC5399220

[38] McCartney M. Margaret McCartney: Game on for Pokemon Go. BMJ. 2016;354:i4306 doi: 10.1136/bmj.i4306 27510374 10.1136/bmj.i4306

[39] Hamrik Z, Sigmundova D, Kalman M, Pavelka J, Sigmund E. Physical activity and sedentary behaviour in Czech adults: results from the GPAQ study. Eur J Sport Sci. 2014;14(2):193–8. doi: 10.1080/17461391.2013.822565 23889330 10.1080/17461391.2013.822565 PMC3935222

[40] Shephard R, Vuillemin A. Limits to the measurement of habitual physical activity by questionnaires. Br J Sports Med. 2003;37(3):197–206. doi: 10.1136/bjsm.37.3.197 12782543 10.1136/bjsm.37.3.197 PMC1724653

[41] Pourmand A, Lombardi K, Kuhl E, O'Connell F. Videogame-related illness and injury: a review of the literature and predictions for Pokemon GO! Games Health J. 2017;6(1):9–18. doi: 10.1089/g4h.2016.0090 28135114 10.1089/g4h.2016.0090

[42] Fox News. Missouri police say 4 teens used 'Pokemon Go' to rob people. 2016 Jul 11. [cited 12 August 2016]. In: Fox News [Internet]. Available from: http://www.foxnews.com/us/2016/07/11/missouri-police-say-4-teens-used-pokemon-go-to-rob-people.html

[43] Griffin A. Pokemon Go: teenager shot dead while hunting creatures. Independent. 2016 Jul 20. [cited 12 August 2016]. Available from: http://www.independent.co.uk/life-style/gadgets-and-tech/news/pokemon-go-death-guatemala-shot-danger-safety-dead-a7145836.html

[44] Joseph B, Armstrong DG. Potential perils of peri-Pokemon perambulation: the dark reality of augmented reality? Oxf Med Case Reports. 2016;2016(10). doi: 10.1093/omcr/omw080 27713831 10.1093/omcr/omw080 PMC5050458

