# Supplementary material for: Gaming behaviour with Pokémon GO and physical activity: A preliminary study with medical students in Thailand
Source: PLoS One. 2018 Jun 29;13(6):e0199813. doi: 10.1371/journal.pone.0199813 (PMC6025865; doi:10.1371/journal.pone.0199813)
Supplement: S1 File — (DOCX) [file pone.0199813.s001.docx]

**Survey questions in Thai language**

**คำชี้แจง** แบบสอบถามประกอบด้วย 3 ส่วน

กรุณาเติมคำในช่องว่างหรือเลือกคำตอบที่ตรงกับตัวท่าน

**แบบสอบถามส่วนที่ 1 ข้อมูลทั่วไป จำนวน 10 ข้อ**

1. รหัสนักศึกษา_______________________

2. สถานที่เรียน  มหาวิทยาลัยวลัยลักษณ์  โรงพยาบาลตรัง  โรงพยาบาลวชิระภูเก็ต

3. ชั้นปี  ปรีคลินิก  คลินิก

4. เพศ  ชาย  หญิง

5. วันเกิด (วัน/เดือน/พ.ศ.) __/__/____

6. น้ำหนัก (กิโลกรัม) ___._

7. ความสูง (เซนติเมตร) ___._

8. รายรับ (บาทต่อเดือน)  < 5,000  5,000 – 9,999  10,000 -14,999  15,000 – 19,999  ≥ 20,000

9. สถานที่พัก (ที่อาศัยเป็นหลัก)  ในมหาวิทยาลัย/โรงพยาบาล  นอกมหาวิทยาลัย/โรงพยาบาล

10. โรคประจำตัว  ไม่มี  มี (โปรดระบุ)

**แบบสอบถามส่วนที่ 2 ข้อมูลการเล่นเกม Pokémon GO จำนวน 6 ข้อ**

1. ท่านเล่นเกม Pokémon GO จำนวนกี่วัน ต่อ สัปดาห์ _ วัน

2. ท่านเล่นเกม Pokémon GO ในแต่ละวันโดยเฉลี่ยเป็นเวลานานเท่าไร __ ชั่วโมง __ นาที

3. ท่านเล่นเกม Pokémon GO ต่อเนื่องแต่ละครั้งโดยเฉลี่ยเป็นเวลาเท่าไร __ ชั่วโมง __ นาที

4. เหตุผลที่ท่านเล่นเกม Pokémon GO (ตอบได้มากกว่า 1 เหตุผล)

 ฆ่าเวลา/ลดความเบื่อ  มีความสนุกสนาน

 ผ่อนคลาย/ลดความเครียด  กระตุ้นสมอง

 มีปฏิสัมพันธ์กับสังคม  เป็นการท้าทาย

 รู้สึกตื่นเต้น  ออกกำลังกาย

 ทำสิ่งที่เป็นไปไม่ได้  เรียนรู้

 อื่นๆ (ระบุ) ____________________

5. ท่านมีวิธีการอย่างไรในการเคลื่อนที่เพื่อทำตามคำสั่งหรือเงื่อนไขของเกม (ตอบได้มากกว่า 1 วิธี)

 เดิน  วิ่ง  ปั่นจักรยาน

 ขับขี่/โดยสารรถจักรยานยนต์  ขับขี่/โดยสารรถยนต์

6. ท่านมีอาการผิดปกติหรือบาดเจ็บหรืออุบัติเหตุที่เกี่ยวข้องกับการเล่นเกม Pokémon GO หรือไม่

 ไม่มี

 มี (โปรดเลือก)

 ปวดเมื่อยกล้ามเนื้อ  หกล้ม

 อุบัติเหตุจราจร  ถูกทำร้ายร่างกาย

 ชัก  วิงเวียน

 อื่นๆ (ระบุ) ____________________

**แบบสอบถามส่วนที่ 3 ข้อมูลกิจกรรมทางกาย จำนวน 16 ข้อ**

| **กิจกรรมการทำงาน** | | |
| --- | --- | --- |
| ต่อไปนี้เป็นคำถามเกี่ยวกับการทำงาน เช่น การเรียน/การอบรม กิจกรรมการทำงานบ้าน การเพาะปลูกและเก็บเกี่ยว การหาปลา/หาอาหาร การรับจ้างทำงานทั่วๆไป เป็นต้น **ในสัปดาห์หนึ่งๆ** | | |
| **คำถาม** | **กิจกรรมที่ทำ** | **คำตอบ** |
| 1 | ท่านทำงานออกแรง/ออกกำลัง**ระดับหนัก** ซึ่งทำให้หายใจแรงและเร็วกว่าปกติมากหรือหอบ ติดต่อกันเป็นเวลานานอย่างน้อย 10 นาที เช่น การยกหรือแบกของหนักๆ การขุดดิน งานก่อสร้าง เป็นต้น | - ใช่ - ไม่ใช่   (**ไม่ใช่** ให้ข้ามไปตอบ ข้อ 4) |
| 2 | โดยปกติ**ท่านทำงานออกแรง/ออกกำลังระดับหนัก**  จำนวนกี่วันต่อสัปดาห์ | จำนวน…..........วัน ต่อสัปดาห์ |
| 3 | โดยปกติ**ท่านทำงานออกแรง/ออกกำลังระดับหนัก**  ในแต่ละวัน เป็นเวลานานเท่าไร  (เฉพาะงานที่ทำติดต่อกันนาน 10 นาทีขึ้นไป) | จำนวน  …………........ชั่วโมง  ……….....….. นาที |
| 4 | **ท่านทำงานออกแรง/ออกกำลังระดับปานกลาง** ซึ่งทำให้หายใจเร็วขึ้นพอควร แต่ไม่ถึงกับหอบติดต่อกันเป็นเวลานานอย่างน้อย 10 นาที เช่น การก้าวเดินเร็ว ๆหรือการยกถือของเบาๆ เป็นต้น | - ใช่ - ไม่ใช่   (**ไม่ใช่** ให้ข้ามไปตอบ ข้อ 7) |
| 5 | โดยปกติ**ท่านทำงานออกแรง/ออกกำลังระดับปานกลาง**  จำนวนกี่วันต่อ สัปดาห์ | จำนวน…..........วัน ต่อสัปดาห์ |
| 6 | โดยปกติ**ท่านทำงานออกแรง/ออกกำลังระดับปานกลาง**  ในแต่ละวัน เป็นเวลานานเท่าไร  (เฉพาะงานที่ทำติดต่อกันนาน 10 นาทีขึ้นไป) | จำนวน  …………........ชั่วโมง  ……….....….. นาที |

| **กิจกรรมการเดินทาง** | | |
| --- | --- | --- |
| ต่อไปนี้เป็นคำถามเกี่ยวกับ**การเดินทาง**ที่ทำเป็นประจำ เช่น ไปทำงาน ไปตลาด ไปซื้อข้าว-ของ ไปวัด-โบสถ์ เป็นต้น **ในสัปดาห์หนึ่งๆ** | | |
| **คำถาม** | **กิจกรรมที่ทำ** | **คำตอบ** |
| 7 | **ท่านเดินหรือถีบจักรยานจากที่หนึ่งไปยังอีกที่หนึ่งติดต่อกันเป็นเวลานานอย่างน้อย 10 นาที** ใช่หรือไม่ | - ใช่ - ไม่ใช่   (**ไม่ใช่** ให้ข้ามไปตอบข้อ 10) |
| 8 | โดยปกติ**ท่านเดินหรือถีบจักรยานจากที่หนึ่งไปยังอีกที่หนึ่งติดต่อกันเป็นระยะเวลาอย่างน้อย 10 นาที**  จำนวนกี่วันต่อสัปดาห์ | จำนวน…..........วัน ต่อสัปดาห์ |
| 9 | โดยปกติ**ท่านเดินหรือถีบจักรยาน** ในแต่ละวัน  เป็นเวลานานเท่าไร  (เฉพาะที่ทำติดต่อกันนาน 10 นาทีขึ้นไป) | จำนวน  …………........ชั่วโมง  ……….....….. นาที |

| **กิจกรรมที่ทำในเวลาว่างเพื่อพักผ่อนหย่อนใจ/นันทนาการ** | | |
| --- | --- | --- |
| คำถามต่อไปนี้เป็นคำถามเกี่ยวกับการเล่นกีฬา การเล่นฟิตเนส และกิจกรรมนันทนาการที่ทำเพื่อความบันเทิง หรือ เพื่อผ่อนคลายในเวลาที่ว่างจากการทำงาน **ในสัปดาห์หนึ่งๆ** | | |
| **คำถาม** | **กิจกรรมที่ทำ** | **คำตอบ** |
| 10 | **ท่านเล่นกีฬา ออกกำลังกายหรือทำกิจกรรมในเวลาว่างเพื่อความบันเทิงหรือพักผ่อนหย่อนใจในระดับหนัก**ติดต่อกันเป็นเวลานานอย่างน้อย 10 นาที ซึ่งทำให้หายใจแรงและเร็วกว่าปกติมาก หรือทำให้หอบ (เช่น เต้นแอโรบิก การวิ่งเหยาะ การว่ายน้ำ การเล่นกีฬา หรือถีบจักรยานเร็วๆ) | - ใช่ - ไม่ใช่   (**ไม่ใช่** ให้ข้ามไปตอบข้อ 13) |
| 11 | โดยปกติ**ท่านเล่นกีฬา ออกกำลังกายหรือทำกิจกรรมในเวลาว่างเพื่อความบันเทิงหรือพักผ่อนหย่อนใจระดับหนัก**  เป็นจำนวนกี่วันต่อสัปดาห์ | จำนวน…..........วัน ต่อสัปดาห์ |
| 12 | โดยปกติ**ท่านเล่นกีฬา ออกกำลังกายหรือทำกิจกรรมในเวลาว่างเพื่อความบันเทิงหรือพักผ่อนหย่อนใจระดับหนัก** เป็นเวลานานเท่าไร (เฉพาะงานที่ทำติดต่อกันนาน 10 นาทีขึ้นไป) | จำนวน  …………........ชั่วโมง  ……….....….. …นาที |
| 13 | **ท่านเล่นกีฬา ออกกำลังกายหรือทำกิจกรรมในเวลาว่างเพื่อความบันเทิงหรือพักผ่อนหย่อนใจระดับปานกลาง**ติดต่อกันเป็นเวลานานอย่างน้อย 10 นาที ซึ่งทำให้หายใจเร็วขึ้นพอควรไม่ถึงกับหอบ (เช่น การยกของที่น้ำหนักเบา ถีบจักรยานไปเรื่อยๆ หรือเล่นเทนนิสคู่) | - ใช่ - ไม่ใช่   (**ไม่ใช่** ให้ข้ามไปตอบ ข้อ 16) |
| 14 | โดยปกติ**ท่านเล่นกีฬา ออกกำลังกายหรือทำกิจกรรมในเวลาว่างเพื่อความบันเทิงหรือพักผ่อนหย่อนใจระดับปานกลาง**  เป็นจำนวนกี่วันต่อสัปดาห์ | จำนวน…..........วัน ต่อสัปดาห์ |
| 15 | โดยปกติ**ท่านเล่นกีฬา ออกกำลังกายหรือทำกิจกรรมในเวลาว่างเพื่อความบันเทิงหรือพักผ่อนหย่อนใจ**ระดับปานกลาง  ในแต่ละวัน เป็นเวลานานเท่าไร | จำนวน  …………........ชั่วโมง  ……….....….. …นาที |

| **กิจกรรมที่เป็นการนั่ง** | | |
| --- | --- | --- |
| ต่อไปนี้เป็นคำถามเกี่ยวกับการนั่ง หรือการนั่ง ๆ นอน ๆ ที่บ้าน หรือ ณ ที่ใด ๆ จะเป็นการนั่งเพื่อเดินทางไปในที่ต่าง ๆ หรือ การนั่งพูดคุยกับเพื่อน นั่งทำงาน นั่งดูโทรทัศน์ แต่ไม่รวมเวลาที่ใช้ในการนอน **ในสัปดาห์หนึ่งๆ** | | |
| **คำถาม** | **กิจกรรมที่ทำ** | **คำตอบ** |
| 16 | โดยปกติในแต่ละวัน **ท่านใช้เวลานั่งเอนกาย**รวมแล้ว  เป็นเวลานานเท่าไร  (เฉพาะที่ทำติดต่อกันนาน 10 นาทีขึ้นไป) | จำนวน  …………........ชั่วโมง  ……….....….. …นาที |

**Survey questions in English language**

**Section 1: Demographic characteristics**

1. Student ID_______________________

2. Campus  Nakhon Si Thammarat  Trang  Phuket

3. Level of education  Preclinical  Clinical

4. Sex  Male  Female

5. Date of Birth __/__/____

6. Weight (kg) ___._

7. Height (cm) ___._

8. Allowance (Baht/month)  < 5,000  5,000 – 9,999  10,000 -14,999  15,000 – 19,999  ≥ 20,000

9. Place of living  On campus  Off campus

10. Underlying illness  No  Yes (please specify)

**Section 2: Patterns of and reasons for playing Pokémon GO and game-related injuries**

1. How many days in a week do you play Pokémon GO? _ day(s)

2. How long do you play Pokémon GO per day? __ hour(s) __ minute(s)

3. How long do you play Pokémon GO per bout? __ hour(s) __ minute(s)

4. What are your reasons for playing Pokémon GO? (select 1 or more answers)

 Pass time/boredom  Have fun

 Relax/de-stress  Keep mind active

 Social interaction  Be challenged

 Feel excitement  Exercise

 Do the impossible  Learn

 Other (please specify) ____________________

5. What are your commuting modes to play Pokémon GO? (select 1 or more answers)

 Walking  Running  Cycling

 Riding a motorcycle  Riding a car

6. Do you have any abnormalities, injuries or accidents related to playing Pokémon GO?

 No

 Yes (select 1 or more answers)

 Muscle strain  Fall

 Traffic accident  Body assualt

 Seizure  Dizziness

 Other (please specify) ____________________

**Section 3: Physical activity participation** (from Global Physical Activity Questionnaire, GPAQ)

| **Activity at work** | | | |
| --- | --- | --- | --- |
| **1** | Does your work involve vigorous-intensity activity that causes large increases in breathing or heart rate like *[carrying or lifting* *heavy loads, digging or construction work*] for at least 10 minutes continuously? | Yes 1  No 2 If No, go to P 4 | P1 |
| 2 | In a typical week, on how many days do you do vigorous-intensity activities as part of your work? | Number of days └─┘ | P2 |
| 3 | How much time do you spend doing vigorous-intensity activities at work on a typical day? | Hours : minutes └─┴─┘: └─┴─┘  hrs mins | P3  (a-b) |
| 4 | Does your work involve moderate-intensity activity that causes small increases in breathing or heart rate such as brisk walking *[or carrying light loads*] for at least 10 minutes continuously?  *[INSERT EXAMPLES] (USE SHOWCARD)* | Yes 1  No 2 If No, go to P 7 | P4 |
| 5 | In a typical week, on how many days do you do moderate-intensity activities as part of your work? | Number of days └─┘ | P5 |
| 6 | How much time do you spend doing moderate-intensity activities at work on a typical day? | Hours : minutes └─┴─┘: └─┴─┘  hrs mins | P6  (a-b) |
| **Travel to and from places** | | | |
| 7 | Do you walk or use a bicycle (*pedal cycle*) for at least 10 minutes continuously to get to and from places? | Yes 1  No 2 If No, go to P 10 | P7 |
| 8 | In a typical week, on how many days do you walk or bicycle for at least 10 minutes continuously to get to and from places? | Number of days └─┘ | P8 |
| 9 | How much time do you spend walking or bicycling for travel on a typical day? | Hours : minutes └─┴─┘: └─┴─┘  hrs mins | P9  (a-b) |
| 10 | Do you do any vigorous-intensity sports, fitness or recreational (*leisure*) activities that cause large increases in breathing or heart rate like [*running or football,]* for at least 10 minutes continuously?  *[INSERT EXAMPLES] (USE SHOWCARD)* | Yes 1  No 2 If No, go to P 13 | P10 |
| 11 | In a typical week, on how many days do you do vigorous-intensity sports, fitness or recreational (*leisure*) activities? | Number of days └─┘ | P11 |
| 12 | How much time do you spend doing vigorous-intensity sports, fitness or recreational activities on a typical day? | Hours : minutes └─┴─┘: └─┴─┘  hrs mins | P12  (a-b) |
| 13 | Do you do any moderate-intensity sports, fitness or recreational *(leisure*) activities that causes a small increase in breathing or heart rate such as brisk walking*,*(*cycling, swimming, volleyball*)for at least 10 minutes continuously?  *[INSERT EXAMPLES] (USE SHOWCARD)* | Yes 1  No 2 If No, go to P 16 | P13 |
| 14 | In a typical week, on how many days do you do moderate-intensity sports, fitness or recreational (*leisure*) activities? | Number of days └─┘ | P14 |
| 15 | How much time do you spend doing moderate-intensity sports, fitness or recreational (*leisure*) activities on a typical day? | Hours : minutes └─┴─┘: └─┴─┘  hrs mins | P15  (a-b) |
| **Sedentary behaviour** | | | |
| 16 | How much time do you usually spend sitting or reclining on a typical day? | Hours : minutes └─┴─┘: └─┴─┘  hrs mins | P16  (a-b) |
